# Structural distress: experiences of moral distress related to structural stigma during the COVID-19 pandemic

**DOI:** 10.1007/s40037-021-00663-y

**Published:** 2021-04-29

**Authors:** Javeed Sukhera, Chetana Kulkarni, Taryn Taylor

**Affiliations:** 1 Departments of Psychiatry/Paediatrics and Centre for Education Research and Innovation, Schulich School of Medicine and Dentistry, Western University, London, Ontario Canada; 2 Hospital for Sick Children (SickKids), Department of Psychiatry, University of Toronto, Toronto, Canada; 3 Department of Obstetrics and Gynecology and Centre for Education Research and Innovation, Schulich School of Medicine and Dentistry, Western University, London, Ontario Canada

**Keywords:** COVID-19, Moral distress, Stigma, Marginalized populations, Professional identity formation

## Abstract

**Introduction:**

The COVID-19 pandemic has taken a significant toll on the health of structurally vulnerable patient populations as well as healthcare workers. The concepts of structural stigma and moral distress are important and interrelated, yet rarely explored or researched in medical education. Structural stigma refers to how discrimination towards certain groups is enacted through policy and practice. Moral distress describes the tension and conflict that health workers experience when they are unable to fulfil their duties due to circumstances outside of their control. In this study, the authors explored how resident physicians perceive moral distress in relation to structural stigma. An improved understanding of such experiences may provide insights into how to prepare future physicians to improve health equity.

**Methods:**

Utilizing constructivist grounded theory methodology, 22 participants from across Canada including 17 resident physicians from diverse specialties and 5 faculty members were recruited for semi-structured interviews from April–June 2020. Data were analyzed using constant comparative analysis.

**Results:**

Results describe a distinctive form of moral distress called structural distress, which centers upon the experience of powerlessness leading resident physicians to go above and beyond the call of duty, potentially worsening their psychological well-being. Faculty play a buffering role in mitigating the impact of structural distress by role modeling vulnerability and involving residents in policy decisions.

**Conclusion:**

These findings provide unique insights into teaching and learning about the care of structurally vulnerable populations and faculty’s role related to resident advocacy and decision-making. The concept of structural distress may provide the foundation for future research into the intersection between resident well-being and training related to health equity.

## Introduction

The global COVID-19 pandemic has had a disproportionately negative impact on the health and well-being of structurally vulnerable and stigmatized groups [1]. A particularly insidious form of inequity is structural stigma. Stigma typically refers to both attitudes and behaviours within and among individuals, whereas structural stigma refers to how such attitudes and behaviours are reflected within cultural norms and organizational policy [2]. Structural stigma disproportionately impacts vulnerable and marginalized groups within healthcare, often resulting in inequitable outcomes. Akin to systemic racism, understanding structural stigma requires deconstructing how individual-level practices are shaped by broader forces [3]. Structural stigma tends to be under-researched compared to other forms of stigma that may be more interpersonal.

The COVID-19 pandemic has also exposed the pervasiveness and consequences of moral distress for healthcare workers. Moral distress (MD) is a concept initially derived from nursing literature that refers to an individual’s reaction when they believe to know the right thing to do but are unable to do it [4]. Moral distress can arise when a sense of responsibility cannot be acted upon due to personal constraints or external barriers. Health professionals can experience moral distress when their values conflict with their workplace. The consequences of moral distress can be lasting [5, 6] and include feelings of powerlessness, sadness, anger, frustration [7], and burnout [8]. There are several other terms that are relevant to the phenomenon of moral distress. For example, moral injury refers to how bearing witness to morally distressing situations can trigger a reaction akin to psychological trauma [9]; moral residue refers to the leftover consequences of moral distress, which might lead to a cumulative negative impact over time [10–12]; while *moral courage* refers to the ability to rise above fear and act based on one’s beliefs [13, 14].

Despite a proliferation of research into moral distress among healthcare workers, the topic remains generally under-explored amongst medical learners. A review by Lamiani and colleagues showed that only 3% of studies explored moral distress among medical learners [15], even though moral distress has been shown to be widely prevalent in this group [16]. Existing research suggests that moral distress among healthcare staff may manifest through communication challenges, care for severe/complex illness, and lack of appropriate care or resources [17]. Generally, there is higher moral distress when there are structural issues with care [18], yet moral distress has rarely been explored in relation to the care of structurally stigmatized individuals and populations.

Resident physicians are uniquely situated to provide particular insights into the topic of structural stigma and moral distress in the context of a public health emergency because of their dual role in healthcare organizations as both health workers and learners [19]. Residents are also in a distinct position in regard to the power dynamics within hierarchical organizations where they tend to be reluctant to challenge authority [20]. During the COVID-19 pandemic, resident physicians were frequently redeployed to provide clinical coverage [21], raising questions of potential moral and ethical implications of their role in healthcare organizations [22] during an emergency situation. In addition, a better understanding of resident perspectives may inform how the clinical learning environment can better prepare future physicians to address health disparities [23]. Thus, we sought to explore how residents perceive moral distress as it relates to the care of structurally stigmatized patients during the COVID-19 pandemic, which is critical to inform our existing understanding of how to improve health equity as well as the well-being of health workers.

## Methods

### Approach

We utilized constructivist grounded theory (CGT) to conduct our study as we worked upon existing research to build theory towards a social process that is not well explained by an established theoretical construct [24]. In this study, we defined moral distress as an emotional state that arises from a situation where an individual feels a tension between what they believe is the best course of action and the best course of action possible [4]. We defined structural stigma as societal-level conditions, cultural norms, and institutional practices that constrain the opportunities, resources, and well-being for stigmatized populations [2].

### Data collection

We electronically shared recruitment notices using social media (Twitter). Consistent with our research question, we sought a theoretical sample that included a diverse group of trainees from different specialties and geographic sites across Canada. Since our focus was on how moral distress manifests in the context of the pandemic, we recruited residents who had worked in a clinical role at a site where patients were admitted to inpatient hospital care with COVID-19 as of March 15, 2020. We excluded any residents who were not working in a clinical capacity or working in a setting where there were no COVID-19 admissions. This was a deliberate decision to address the potential of geographic variation in pandemic response. We included this exclusion criteria in an effort to ensure that only those residents who worked in inpatient settings where COVID-19 patients were hospitalized participated in our study. We also chose to exclude any residents from the geographic location where the principal investigator is based. This was a deliberate decision as part of our approach to being critically reflexive. Interviews took place over a 90-day period between April and June during the first phase of the COVID-19 pandemic in Canada. Our local University Research Ethics Board (at Western University) provided approval to conduct the study (REB#115763).

Utilizing a theoretical sampling strategy, we successfully recruited 17 resident and 5 faculty participants. Of the participants, 19 identified as female and 4 as male. Eight different residency programs were represented in the sample from across Canada including representation from diverse geographical regions including urban and rural sites. Most residents were either PGY1 (postgraduate year one), or PGY3, with a few in PGY2, PGY4, or PGY5. Most participants identified as working in internal medicine. The second most common specialties were psychiatry and family medicine, followed by obstetrics and gynecology, emergency medicine, and anesthesia.

Participants took part in 30-to-90-minute, semi-structured interviews via teleconference. Interviews were recorded and transcribed verbatim. Questions focused on experiences of moral distress in relation to structural stigma, examples of these experiences, and how residents sought to reconcile their distress. Consistent with CGT methodology, the discussion guide was revised iteratively in response to preliminary analysis [24]. We therefore revised the discussion guide accordingly by exploring more details about how residents reconcile moral distress, and the role of faculty. We expanded our sample to include faculty who provided clinical supervision for residents during the pandemic.

### Analysis, team composition, and reflexivity

Once transcribed, coding and analysis were primarily conducted by the principal investigator utilizing constant comparative analysis [24] to develop focused codes, relating codes to one another, and working towards the development of an explanatory theory. Data collection continued until sufficient data was collected to enable a coherent and logical conceptual understanding of the process under study [25]. A synthesis of results was shared with participants via email to ensure the results were consistent with the research questions and their experience and perspectives.

Team member composition included the principal investigator (JS), who is a child and adolescent psychiatrist, faculty member and PhD scientist in health professions education, as well as co-investigator (TT), a PhD scientist in health professions education and practicing obstetrician/gynecologist. The team also included co-investigator (CK), who is a child and adolescent psychiatrist involved in residency education at a different site than JS and TT. All three members worked as physicians in hospital-based and academic settings. During analysis, JS shared key codes and representative quotations on a regular basis with the team during regular analytical meetings.

## Results

### How and why residents experienced moral distress due to structural stigma

Participants described several different examples of how and why they experienced moral distress. Certain stigmatized patient groups were perceived as being disproportionately impacted by policy decisions during the pandemic, and there was considerable distress related to potential harm experienced by these groups. Specifically, participants were distressed when they witnessed the adverse impact of structural stigma through restrictive visitor policies, through limited access to culturally and linguistically appropriate services, on individuals with cognitive impairment, and through the disproportionate impact of the pandemic on individuals who needed mental health and/or addictions care. One participant noted that the pandemic has *“uncovered some of the weaknesses of the armor in our system,”* going on to say that the pandemic pointed out gaps for *“patients who are isolated or who have language barriers”* (R04).

Most participants were distressed that structural stigma embedded within policy decisions contributed to compromised care for structurally vulnerable patient groups. One participant noted how lack of proper interpretation services can lead to a *“delay in diagnosis or misdiagnosis,”* or *“the patient not understanding what their condition is or what their plans are leading to non-adhering to medication, missed follow-up appointments … or increased morbidity”* (R05). Another resident stated, *“It makes me think why are we in this situation where you have to be in a situation where you have to be English speaking, have perfect hearing to get good quality care”* (R16).

In general, moral distress was experienced as powerlessness. The nature of the pandemic seemed to amplify distress due to a sense of urgency, leading to changes in the distribution of power and how policy decisions were made. Residents described feeling that pandemic policy was quickly developed within hierarchical power structures at a *“higher level”* (R02) and that they did not have much of a say in decision making. One participant described how they felt they were getting *“edicts coming down from much higher,”* as they were told *“this is the new rule, and you have no input” *(R03). Another noted that in the context of a pandemic, *“there’s not really space for feedback”* (R02).

A prominent influence on how residents perceived moral distress was how they felt faculty responded. Faculty responses appeared to mediate residents’ experience of moral distress by either enhancing residents’ agency or invalidating their concerns. One resident (R04) described how *“transparency and honesty”* from their program director helped them feel supported. A faculty participant also recognized that they play an important role in helping residents, stating that when faculty in program leadership are *“openly trying to create a culture of inclusion or culture of transparency it makes such a difference,”* (F05). Our analysis suggested that a key variable in whether residents perceived faculty as supportive or not was whether faculty enhanced the influence of the residents to make change or acted in ways that led them to feel more powerless. For example, faculty that were honest and open about their own limitations and transparent about how decisions were made were perceived as helpful. Many residents also appreciated exemplary faculty role models who recognized structural aspects of care by advocating *“behind the scenes”* (R15, R16).

### How residents sought to reconcile or respond to their moral distress

Resident participants attempted to reconcile their moral distress though sharing, advocacy, and by *“doing extra.”* They sought support from one another and attempted to practice acceptance while making meaning out of their experiences. Being able to debrief with peers who are in *“similar positions”* was viewed as an *“important coping mechanism”* (R15). Some participants also described how their moral distress led to a sense of self-blame and helplessness which then led them to work to accept that there is *“only so much that I as one person can do”* (R06). One resident described this experience as reminding themselves that *“… I can only be as good of a doctor as I try to be … sometimes you fail no matter how hard you try and that’s okay because you don’t see how many people you’ve helped” *(R15).

Many residents employed advocacy as a strategy to reconcile moral distress. A resident stated that advocacy enabled them to* “translate something that felt unfair and wrong into action that was productive”* (R16). Advocacy was targeted both at the patient level and as a way to inform policy decisions within organizations. For example, one resident participant noted that they created an *“advocacy group”* to address the disproportionate impact of pandemic policy on women’s reproductive health (R14), while another faculty participant described how residents were consistently going *“the extra mile”* by advocating for their *“individual patients and families”* that experienced structural stigma (F01). Perceptions of power also shaped residents’ approach to advocacy. Feelings of powerlessness inspired some residents to *“think very creatively about ways of delivering outstanding patient care”* to address structural barriers to stigmatized populations (R09). Advocacy was also fueled by a sense of *“pressure”* regarding the potential of adverse outcomes for stigmatized patients (R07). A resident stated they felt *“more of a sense of urgency”* because *“in a pandemic there is a sense of urgency that this is going to cause this effect …. and if I don’t fix it more people will get hurt or die.” *(R13).

Although many resident participants noted that they were comfortable speaking up about structural stigma during the pandemic, they also described a tension between speaking up and staying silent. This tension stemmed from their perception of a power differential and fear of retribution for their advocacy. One resident stated they would be *“hesitant”* to approach *“higher ups”* (R1), while several others noted that the dynamics during the pandemic made it harder to speak up. One stated,

*… I think also *[of] *this like *[a] *cultural narrative … of an army fighting an invader is … very much designed to not ask questions, to stick with the hierarchy, to do what you’re said, to do what you are told. Medicine does have a strong hierarchy and I think that hierarchy has become much more rigid in a pandemic … and if you dissent or if you have questions … “you’re against us”. *(R02)

Faculty participants agreed that fear of retribution for *“speaking up”* is not unfounded (F04). One faculty participant acknowledged that residents may get punished for speaking up in *“subtle ways”* like not getting the vacation time or rotation that they wanted (F05).

Due to the perception that advocacy would need to be constrained and tempered due to the power differential that residents experienced, many sought strategies to speak up about inequity that attempted to strike a balance. This was described by one participant as *“writing in the margins”* (R04). The idea of writing in the margins was also described by another participant who stated that although they feel powerless, they know there are *“certain things that we kind of do to try to get around that system”* (R11). Another participant described their approach as *“asking a lot of questions … rather than with guns blazing”* because of experiences where they were reprimanded for bringing up *“equity and social justice”* as a medical student (R3). Others described similar experience of being afraid to speak up because they did not want to be admonished as a *“social justice butterfly”* by their preceptor or questioning their own expertise and doubting their experiences leading them to *“constantly struggle between speaking out or staying quiet”* (R05). Similarly, other residents stated *“sometimes I stay quiet”* because they do not feel they have any influence or can make a difference by speaking up or advocating (R10, R12, R17).

Generally, residents also sought to reconcile their moral distress by going above and beyond the call of duty. This was described by most participants as *“doing extra.”* The idea of doing extra seemed to be a natural response to feeling helpless. By doing things they felt were within their domain of influence, residents felt less helpless. One described this by saying that they worked extra hard to keep vulnerable patients linked to families, noting that doing extra helped them *“with the sensation of feeling a little bit helpless”* (R06). Similarly, there were examples of residents who noted that they felt a *“sense of responsibility”* to contribute which gave them a sense of *“meaning and purpose”* (R09). Throughout the interviews, there were many examples of how residents went above and beyond what was expected of them. One participant described a situation by sharing how they were caring for a patient from a foreign country without any family members. The resident stated they were *“advocating quite strongly”* because they were distressed by the fact that the patient did not have a place to live or family supports, stating *“it created ‘a lot of tension’ for them”* (R07).

### Moral residue and consequences of moral distress

Although resident participants often sought ways that they could *“go the extra mile”* or do more than was expected of them, they also appreciated that doing extra could also take its own toll and potentially worsen their distress. We use the term moral residue to capture these consequences.

For example, one resident who spent extra time in a vulnerable patient’s room described how they experienced compassion fatigue as a consequence of feeling like they needed to be *“emotionally attuned”* to stigmatized patients stating, *“… just like giving so much of ourselves to patients in a way that can be draining”* (R01). Others noted that although they found it *“gratifying”* to spend extra time with families, it can be *“exhausting”* to answer so many questions (R06). The risk of burnout was summarized by a resident participant who stated that they knew that they can’t *“just keep doing extra”* and that this realization makes them feel *“guilty”* when they set boundaries, wondering if they will reach a point at which doing extra *“doesn’t feel sustainable anymore”* (R16).

Faculty participants also described that residents tend to respond to distress by over-performing. One stated, *“I think … a resident’s response to a challenge is just to work … harder faster stronger better and so I think that’s why … especially during a time like this they can be super high risk for burnout …”* (F01). Another faculty participant noted that the identity of physicians as *“heroes”* lead residents to be *“indoctrinated”* to go beyond appropriate limits and feel guilty for taking care of themselves (F02). This same faculty member suggested that addressing the issue of moral distress required confronting and admitting maladaptive aspects of the culture of medical training stating, *“I think that’s what we need to help residents wrestle with and say, ‘welcome to the medical profession, it is actually a profession that’s founded on moral distress’”* (F02).

## Discussion

**Fig. 1:**
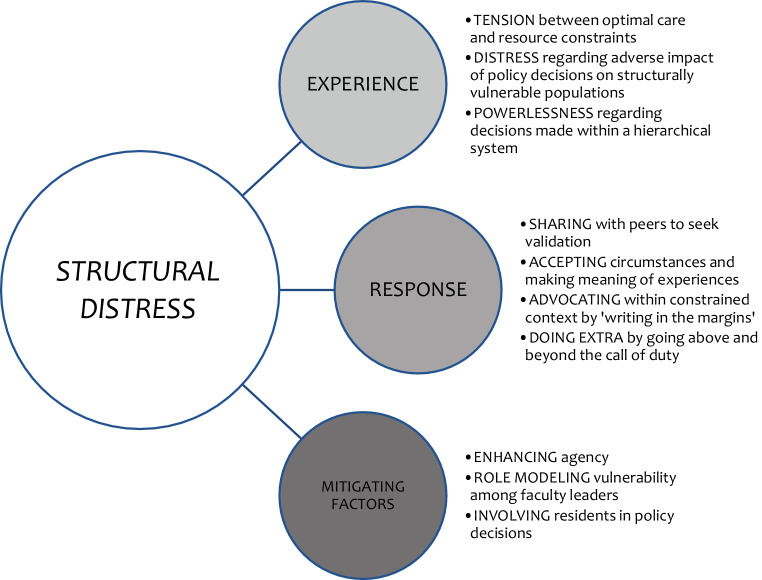
Features of “structural distress”—a unique form of moral distress from a study of Canadian resident physicians’ experiences of moral distress related to structural stigma during the COVID-19 pandemic

Overall, we found that moral distress related to structural stigma was experienced as powerlessness by resident physicians. They sought to reconcile their moral distress by doing extra, even though they recognized that going above and beyond expectations may represent a maladaptive, unsustainable coping strategy with adverse consequences for their mental health. The extent to which faculty mitigated power differential and either enhanced or diminished resident agency was considered as a buffer to residents’ experiences of distress. Our findings provide unique insights into teaching and learning about the care of structurally vulnerable populations and faculty’s role related to resident advocacy and decision-making. A visual depiction of our findings is illustrated in Fig. 1, outlining a unique form of moral distress that we describe as structural distress.

### Powerlessness as a core feature of structural distress

Our findings provide unique insights about how moral distress may manifest as powerlessness. Previous research on moral distress among medical learners has found powerlessness as one component of moral distress [15]; however, our findings suggest that powerlessness is a core feature of moral distress that relates to structurally vulnerable populations.

Research on moral distress suggests that power and hierarchy are important components to consider. For example, moral distress can be perceived differently based on an individual’s place in the organizational hierarchy [26] or due to the power differential between different professions [27]. Other studies suggest power and hierarchy play a role in both moral distress and moral courage [8, 17, 27, 28]. In one study that explored moral courage in nursing students, most students cited power differential, fear of consequences, and lack of confidence as key influences contributing to their hesitation to demonstrate moral courage, often remaining as passive spectators [29]. Our finding that powerlessness was a core feature of moral distress associated with the care of stigmatized populations suggests that further research into the powerlessness associated with moral distress may help improve efforts to advance health equity.

### The consequences of doing extra as a response to structural distress

Our participants appeared to experience powerlessness in the context of witnessing structural inequity; however, they responded by going above and beyond expectations. Many described how advocating for their patients or doing extra helped them cope with their distress by enhancing their sense of agency and control. This finding is consistent with previous research that found those who acted at the time of experiencing distress or something close to it experienced lower distress, while those who expressed regret for their inaction experienced higher distress [18]. In our study, those who did speak up also attempted to reconcile their own perceived powerlessness by advocating within certain constraints. This finding is similar to other research that found individuals’ actions in response to moral distress could include indirectly challenging problematic behaviour [30] or removing themselves from distressing situations to convey disapproval [31, 32].

The finding that resident physicians may be attempting to reconcile their moral distress by doing more than what is expected of them may have implications for resident well-being. Doing extra work has the potential to worsen psychological distress and lead to greater harm [33, 34]. Previous research on moral distress among medical students found that responses to cope with moral distress may be either adaptive or maladaptive. For example, avoidance, moral disengagement, blunting, denial, and detachment may worsen distress and perpetuate poor psychological outcomes [16].

The faculty participants in our study also underscored the problematic nature of the “hero” narrative and professional culture within medicine. One even noted that medicine is a profession that is *“founded”* on moral distress (F02). The culture of medicine, particularly in the context of a pandemic, perpetuates this narrative, with language such as “frontline,” “fight,” and “hero.” There is increasing recognition that such narratives can be harmful to well-being and contribute to alarmingly increasing rates of physician distress and burnout [35, 36].

### Faculty as a buffer to structural distress

Faculty play an important role in buffering against the negative consequences of structural distress. Previous research on moral distress notes that mentoring has a powerful effect on the empathy and may help to protect against cynicism and detachment [37–39]. Our participants noted that faculty mitigated structural distress by either enhancing residents’ agency and sense of power to influence change or invalidated their agency by perpetuating residents’ feelings of powerlessness. There were three ways in which faculty shared power with residents. First, they were transparent about limitations of their own power. They also role modeled vulnerability and acknowledged a sense of shared powerlessness. Finally, they attempted to involve residents as much as possible in making decisions and choosing the course of action they would take in response to their distress.

### Implications

The concept of structural distress provides a novel way of understanding how moral distress related to structural inequity may manifest among medical learners. Shortly after the onset of the COVID-19 pandemic there was widespread recognition that the pandemic was disproportionately affecting structurally vulnerable populations. As academic medicine moves into post-pandemic reality, future research into the novel concept of structural distress will provide additional insights into how the clinical learning environment can adequately prepare future physicians to address health disparities and advance equity for structurally vulnerable populations. Tab. 1 provides a summary of key implications from our study.

**Table 1:** Implications from a study of Canadian resident physicians’ experiences of moral distress related to structural stigma during the COVID-19 pandemic

FINDINGS	EXAMPLES	IMPLICATIONS
**Residents experience structural distress as powerlessness, responding by doing extra and potentially worsening their psychological well-being**	*“I definitely do not think I have a voice …” *(R02) *“I think like a resident’s response to a challenge is just to work like harder faster stronger better and so I think that’s why … they can be super high risk for burnout …” *(F01)	**Programs must draw explicit attention to structural distress as part of residency training** **Critical perspectives on the hero narrative and culture of medical training must be highlighted as part of discussions on structural distress**
**Faculty responses to resident experiences mitigated the adverse impact of structural distress**	*“Faculty ultimately determine what I can do … My decision making is curtailed by … who approves my decisions because I am at such a junior level. Faculty create the norms in which we work.” *(R03) *“We haven’t indoctrinated our faculty to be good faculty members and role models, as a result their residents are bearing the fruits of our failed system on health advocacy …” *(F02)	**Teaching faculty should be prepared to address structural distress and embed advocacy into training** **Faculty must be supported in their own advocacy for themselves and on behalf of residents**
**Structural distress mitigated by enhancing resident agency**	*“I think in general a lot of us like I do feel powerless but there are certain things that we kind of do to try to get around that system.” *(R11)	**Faculty and organizations can enhance resident agency through creating a culture of transparency and involving residents in policy decisions**

### Limitations

The circumstances of the COVID-19 pandemic limited our ability to recruit through traditional means and therefore this study relied solely on social media for recruitment of participants. An additional limitation was the rapidly evolving nature of the pandemic. We recognized during our analysis that interviews conducted in the early phase of the pandemic reflected a distinct level of distress compared to the interviews that were conducted 3–4 months after the pandemic began. Lastly, we limited our recruitment to a Canadian context.

## Conclusion

In this study, we explored how resident physicians experience moral distress related to structural stigma during the COVID-19 pandemic. Results suggest a distinctive form of moral distress which centers upon the experience of powerlessness leading some resident physicians to go above and beyond the call of duty, potentially worsening their psychological well-being. Our findings indicate that faculty play a key role in mitigating the impact of structural distress by role modeling vulnerability and involving residents in policy decisions. Overall, the concept of structural distress provides insights into how residents experience the care of structurally vulnerable populations. Future scholarly work on structural distress should further explore experiences of structural distress among all levels of medical learners.
